# In Vitro Anti-Obesity Effect of Shenheling Extract (SHLE) Fermented with *Lactobacillus fermentum* grx08

**DOI:** 10.3390/foods11091221

**Published:** 2022-04-23

**Authors:** Xian-Tao Yan, Wenmiao Zhang, Yanyan Zhang, Ziqi Zhang, Dawei Chen, Wenqiong Wang, Wenlong Ma, Hengxian Qu, Jian-Ya Qian, Ruixia Gu

**Affiliations:** 1 College of Food Science and Engineering, Yangzhou University, Yangzhou 225012, China; yanyang214@126.com (X.-T.Y.); zwm19962616177@163.com (W.Z.); zzq2549877968@163.com (Z.Z.); dwchen@yzu.edu.cn (D.C.); wenqiong.happy@163.com (W.W.); wenlong-ma@yzu.edu.cn (W.M.); quhengxian@163.com (H.Q.); 2 Jiangsu Key Laboratory of Dairy Biotechnology and Safety Control, Yangzhou 225127, China; 3 Department of Cuisine and Nutrition, Hanshan Normal University, Chaozhou 521000, China; 4 Testing Center of Yangzhou University, Yangzhou 225012, China; zyy@yzu.edu.cn

**Keywords:** anti-obesity, medicine food homology, *Lactobacillus fermentum*, ferment

## Abstract

Obesity is a common global problem. There are many fat-reducing herbal prescriptions in traditional Chinese medicine that have been proven to be safe and functional during long-term application. Microbial fermentation can improve the efficacy of herbal medicine and improve the unsavory flavor. In this study, Shenheling extract (SHLE) composed of six medicine food homology materials was used as the research object. The purpose of this study was to evaluate the effects of *Lactobacillus* *fermentum* grx08 fermentation on the antiobesity efficacy and flavor of SHLE. We found that *L. fermentum* grx08 grew well in SHLE. After 72 h of fermentation, the total polysaccharides, total flavonoids, total polyphenols and total saponins of SHLE decreased, but the lipase inhibitory activity and total antioxidant capacity (FRAP) were significantly increased (*p* < 0.01). There were no significant differences in the α-glucosidase inhibition rate and DPPH· clearance rate before or after fermentation (*p* > 0.05). In addition, the fermentation reduces the unpleasant flavors of SHLE such as bitterness and grassy and cassia flavors. This study demonstrates that SHLE fermented by *L. fermentum* grx08 improved some anti-obesity functions and improved the unpleasant flavor.

## 1. Introduction

Obesity is a common chronic disease worldwide, and it is a risk factor for many chronic diseases such as type 2 diabetes, cardiovascular disease and nonalcoholic fatty liver diseases [1]. According to modern medicine, the main cause of obesity is energy absorption exceeding consumption, accompanied by hyperlipidemia, hyperglycemia, insulin resistance, lipid peroxidation, chronic inflammation and other symptoms [2]. It is difficult to achieve an overall effect with a single targeted therapy in the context of obesity and other chronic diseases with multiple metabolic disorders [3]. The current consensus is that multitarget therapy [4] is suitable for diseases with multiple causes such as obesity. Compound prescriptions of Chinese herbal medicine have the advantage of multiple components and play an omnidirectional regulatory role in the body through multiple targets [5].

In the clinic, there are many classic prescriptions of traditional Chinese medicine, such as Ginseng-plus-Bai-Hu-Tang [6], Huang-Qi San [7], JTTZ [8], Danggui-Baizhu-Tang [9], Bofu-tsusho-san [10,11] and Hwang-Ryun-Haedok-Tang [12], that have shown good weight loss effects. Shenheling (SHL) is a compound formulated according to the theory of weight loss in traditional Chinese medicine. SHL consists of six herbs, namely *Panax ginseng* C. A. Meyer, *Nelumbo nucifera* Gaertn., *Poria cocos* (Schw.) Wolf., *Vigna umbellata* (Thunb.) Ohwi et Ohashi, *Citrus sinensis* (Linn.) Osbeck and *Cinnamomum cassia* Presl. In daily life in China and other Asian countries, they are eaten alone or in combination to make tea or soup. It is also commonly used in dietary supplements in Western countries. These raw materials are all from the list of items that are ‘both food and medicine’ (also known as ‘Medicine and food come from the same source’ or ‘medicine food homology’, MFH) published by the National Health Commission of the People’s Republic of China [13], and their edible safety is well-guaranteed.

Lactic acid bacteria (LAB) have many functional properties and are widely used for weight loss [14]. Some studies have shown that fermentation has a beneficial effect on the anti-obesity properties of Chinese herbal medicine [15,16,17]. However, there are few reports on the use of MFH raw materials to form a compound that is fermented by lactic acid bacteria and then used to lose weight. In this study, *Lactobacillus fermentum* grx08 [18] with the lipid-lowering function was used to ferment SHL extract (SHLE) in order to obtain a functional drink with a stronger weight loss effect. In addition, Shi Yuan et al. [19] found that lactic acid fermentation was an effective method to reduce the off-flavor, such as the “hay”- and “green”-like aroma of pea. Therefore, this study also expects that LAB could remove the grassy and green odors of SHLE.

To explore the comprehensive regulatory effect of lactic acid bacteria fermentation SHLE on obesity and the changes in flavor and other substances, this study selected the inhibition of lipase activity, the inhibition of α-glucosidase activity, the total antioxidant capacity (FRAP method) and the ability to clear DPPH· as the indicators of anti-obesity in vitro, and the compounds present in SHLE before and after the fermentation were analyzed by UPLC-MS/MS and GC-MS.

## 2. Materials and Methods

### 2.1. Chemicals

p-Nitrophenyl palmitate, p-nitrophenyl α-d-glucopyranoside (p-NPG), pancreatic lipase, orlistat, acarbose and 1,1-diphenyl-2-trinitrophenylhydrazine (DPPH) were all purchased from Shanghai Macklin Biochemical Technology Co., Ltd., Shanghai, China. α-Glucosidase, gallic acid, rutin, ginsenoside Re, vitamin C and anhydrous glucose were obtained from Shanghai Yuanye Biotechnology Co., Ltd., Shanghai, China. Folin–Ciocalteu reagent was purchased from Sangon Bioengineering (Shanghai) Co., Ltd., Shanghai, China. The total antioxidant (FRAP) kit was purchased from Nanjing Jiancheng Biological Engineering Research Institute Co., Ltd., Nanjing, China. The rest of the chemicals, reagents, consumables and culture media were purchased from National Pharmaceutical Chemical Reagent Co., Ltd., Shanghai, China.

### 2.2. Composition of SHL and Preparation of SHLE

*Panax ginseng* C. A. Meyer, *Nelumbo nucifera* Gaertn., *Poria cocos* (Schw.) Wolf., *Vigna umbellata* (Thunb.) Ohwi et Ohashi, *Citrus sinensis* (Linn.) Osbeck and *Cinnamomum cassia* Presl. were purchased from Beijing Tongrentang Health Pharmaceutical Co., Ltd., Beijing, P. R. China, and identified by chief pharmacist Ying Yao, Yangzhou University, P. R. China. The voucher specimens, YZU20201015-1~6, were preserved in the specimen room of Yangzhou University, Yangzhou, Jiangsu Province, P.R. China. SHL is composed of six kinds of raw materials, all of which belong to the category of homologous medicinal food, the proportions of which are shown in Table 1. The fully crushed SHL powder and ultrapure water were mixed evenly at a ratio of 1:10 (g/mL), soaked at room temperature for 30 min, heated at 100 °C for 30 min, cooled and centrifuged at 4000× *g* for 10 min. The supernatant was taken as SHLE.

### 2.3. Preparation of Fermented SHLE and Lactobacillus fermentum grx08

*Lactobacillus fermentum* grx08 (CGMCC No. 7695) was provided by the Jiangsu Provincial Key Lab of Dairy Biotechnology and Safety Control, Yangzhou University, China. *L.* *fermentum* grx08 was inoculated into conventional MRS medium and cultured at 37 °C for 18 h. Then, the cells were centrifuged at 5000× *g* for 1 min, the supernatant was poured out, sterile normal saline was added to wash the precipitated bacteria and washing was repeated twice. Finally, the bacterial suspension of *Lactobacillus fermentum* grx08 was obtained by resuspending bacteria in sterile physiological saline and adjusting the OD_600_ to 1.0. The above bacterial suspension was inoculated into SHLE at a ratio of 3% (*v*/*v*) and then cultured under anaerobic conditions at 37 °C for 72 h. Immediately after the inoculation, 10 mL of the mixture was sampled, of which 2 mL was used to count living bacteria and the rest was centrifuged at 5000× *g* for 5 min. The supernatant was taken as the fermentation broth for 0 h and stored at −80 °C until use. After that, samples were obtained and treated according to the above method every 3 h until the end of fermentation at 72 h (each three hours from T0 to T72.).

### 2.4. Determination of Live Bacterial Count, pH and Acidity

The number of living bacteria in the sample was determined by the plate colony counting method. The pH value was measured by an FE20 pH meter (METTLER TOLEDO Int. Ltd., Zurich, Switzerland). The pH meter was calibrated with pH 4.01 and 6.86 standard buffer solutions before use. The determination method of titration acidity was as follows: Weigh a 5 g sample and titrate by 0.1 mol/L NaOH solution until a pH value up to 7.0 is achieved. The number of milliliters of NaOH solution used ×20 is the acidity value of the sample (°T).

### 2.5. Determination of Total Polysaccharides Content (TPSC)

The TPSC in the sample was determined by the phenol–sulfuric acid method and slightly modified according to the method of Nazeam et al. [20], and anhydrous glucose was used as the standard. First, the samples were diluted 50-fold with distilled water. Then, 0.5 mL of the diluted sample, 0.5 mL of 6% phenol solution and 2.5 mL of concentrated sulfuric acid were added, mixed well and allowed to stand at 25 °C for 30 min. Finally, 200 µL was pipetted into a 96-well plate, and the absorbance at 490 nm was measured by a spectrophotometer (Thermo Fisher Co., Ltd., Waltham, MA, USA). The data were expressed as mg of glucose equivalent (GlcE) per mL of SHLE.

### 2.6. Determination of Total Flavonoids Content (TFC)

The TFC was determined by the aluminum nitrate colorimetric method with rutin as the standard and slightly modified according to the method of Chen et al. [21]. The sample (0.5 mL) was mixed with 0.05 g/mL NaNO_2_ (1 mL) and allowed to stand for 6 min. Then, 0.1 g/mL Al(NO_3_)_3_ (1 mL) was added, mixed evenly and allowed to rest for 6 min. Next, 0.04 g/mL NaOH (3 mL) was added, mixed evenly and allowed to rest for 15 min. Finally, 200 µL was pipetted into a 96-well plate, the absorbance at 510 nm was measured by a spectrophotometer (Thermo Fisher Co., Ltd., Waltham, MA, USA) and the data are expressed as milligrams of rutin equivalent (RE) per milliliter of SHLE.

### 2.7. Determination of Total Polyphenols Content (TPC)

TPC was slightly modified according to the literature of Derakhshan et al. [22]. As determined by the Folin–Ciocalteu method, a standard curve was prepared with gallic acid as the standard. A total of 400 µL of Folin–Ciocalteu reagent was added to 100 µL of standard or sample and mixed well. After 1 min, 300 µL of 10% sodium carbonate was added to the mixture, mixed well, brought to a volume of 5 mL with ultrapure water and incubated at room temperature for 60 min. Finally, 200 µL was pipetted into a 96-well plate. The absorbance was measured at 765 nm by a spectrophotometer (Thermo Fisher Co., Ltd., Waltham, USA). Data are expressed as mg of gallic acid equivalent (GAE) per mL of SHLE.

### 2.8. Determination of the Total Saponin Content (TSC)

TSC was determined by the vanillin–sulfuric acid colorimetric method with ginsenoside Re as the standard and slightly modified according to the method of Kamyab et al. [23]. Ten microliters of the sample were placed in a 1.5 mL centrifuge tube and dried at 42 °C for 1 h, and then 100 µL of vanillin (10% *w*/*v*) solution in ethanol was added and vortexed for 30 s. Then, 750 µL of 75% concentrated sulfuric acid was added and mixed well on ice. The mixture was then incubated at 60 °C for 20 min. To stop the reaction, the samples were cooled on ice for 10 min. Finally, the absorbance was measured with a spectrophotometer (Thermo Fisher Co., Ltd., Waltham, MA, USA) at 544 nm. The data were expressed as mg of ginsenoside Re equivalent (GRE) per mL of SHLE.

### 2.9. Determination of Pancreatic Lipase Activity Inhibition

The method mentioned in Kim et al.’s study was slightly modified [24]. In a 280 µL reaction system, 180 µL of pH 7.2 phosphate buffer, 40 µL of sample and 20 µL of 10 mM p-NPP (p-nitrophenyl palmitate) were added in succession and incubated at 37 °C for 10 min, and then 40 µL of 10 mg/mL pancrelipase solution was added, fully mixed and incubated at 37 °C for 15 min, followed by the immediate addition of 200 μL of absolute alcohol to terminate the reaction. The mixture was centrifuged at 10,000× *g* for 2 min, and the supernatant was taken. The absorbance was measured at a wavelength of 405 nm by a spectrophotometer (Thermo Fisher Co., Ltd., Waltham, USA) and recorded as B_1_. The sample was replaced with phosphate buffer as the sample blank control and recorded as B_0_. The sample without inhibitors was taken to measure inhibitor-free activity, denoted A_1_; the corresponding negative control without enzyme was used as a negative control without inhibitor, denoted A_0_. Orlistat was used as a positive control.

Lipase activity inhibition (%) = (1 − (B_1_ − B_0_)/(A_1_ − A_0_)) × 100. (1)

### 2.10. Determination of α-Glucosidase Activity Inhibition

The method mentioned in the literature by Silva et al. was slightly modified [25]. One hundred microliters of pH 6.8 phosphate buffer, 20 μL of sample and 20 μL of 20 mM p-NPG were added to a 96-well plate and incubated at 37 °C for 10 min, and then 20 μL of 1 U/mL α-glucosidase solution was added, mixed well and incubated at 37 °C for 15 min. A spectrophotometer (Thermo Fisher Co., Ltd., Waltham, USA) was immediately used to measure the absorbance at a wavelength of 405 nm and the absorbance was recorded as B_1_. The sample was replaced with phosphate buffer as a sample blank control and recorded as B_0_. The sample without inhibitor was used to measure inhibitor-free activity and recorded as A_1_. Correspondingly, the negative control without inhibitor lacked enzyme and was recorded as A_0_. Acarbose was used as a positive control.

Inhibition of α-glucosidase activity (%) = (1 − (B_1_ − B_0_)/(A_1_ − A_0_)) × 100. (2)

### 2.11. Determination of Total Antioxidant (FRAP) Capacity

Determination of the total antioxidant capacity of the sample by the iron reduction (FRAP) method was carried out according to the kit instructions [26]. Then, 180 µL of FRAP working solution was added to the 96-well plate, and 5 µL of sample or ultrapure water was added to the working solution and mixed. The absorbance was recorded at 593 nm by a spectrophotometer (Thermo Fisher Co., Ltd., Waltham, MA, USA) after incubation at 37 °C for 3 min. The standard curve was determined with FeSO_4_ as the standard product. The unit of the total antioxidant capacity of the sample is expressed in ‘mM FeSO_4_ equivalent’. Vitamin C was used as a positive control.

### 2.12. Determination of Scavenging Ability of DPPH·

The method mentioned in the literature by Kwon et al. was slightly modified [27]. First, the sample was diluted 10-fold, and 45 µL of the diluted sample was added to a 96-well plate. Then, 100 µL of 0.2 mM DPPH solution (in ethanol) was added, mixed well and placed in the dark for 30 min at room temperature. The absorbance A_1_ at 516 nm was measured by a spectrophotometer (Thermo Fisher Co., Ltd., Waltham, MA, USA), and 45 µL of anhydrous ethanol was used to replace the sample as blank absorbance A_0_. Ascorbic acid was selected as the positive control. The unit of the DPPH· scavenging rate is recorded as ‘%’. Vitamin C was used as a positive control.

DPPH·scavenging rate (%) = (A_0_ − A_1_)/A_0_ × 100. (3)

### 2.13. Determination of Volatile Flavor Compounds and Sensory Evaluation

The determination of volatile flavor compounds before and after fermentation was slightly modified according to the method of Dan et al. [28]. Aging of the extraction head: aging occurred at the inlet at 250 °C from 30–60 min. Solid phase microextraction conditions: adsorption on a magnetic stirrer at 45 °C for 60 min. Desorption conditions: desorption for 3 min at 250 °C. Chromatographic conditions were set as follows: carrier gas He, flow rate 1.0 mL/min; splitless injection, inlet temperature 250 °C. The temperature program was set to start at 35 °C, and after being held for 5 min, it was raised to 140 °C at a rate of 5 °C/min, held for 2 min, then raised to 250 °C/min at a rate of 10 °C and held for 3 min. The mass spectrometry conditions were set to full scan mode, EI ion source, electron energy 70 eV, ion source temperature 230 °C, mass scan range *m*/*z*: 35–500 AMU, and no solvent delay. Through the NIST2.2 standard library of the MassHunter workstation that comes with the machine, the mass spectral data of each component were automatically retrieved, and o-chlorobiphenyl was used as the internal standard to calculate the relative content of each component.

Referring to the literature of Vargas-Ramella [29], Ghorbani [30] and Cho [31], the following sensory evaluation method was designed. After training, ten postgraduates majoring in food science and engineering conducted sensory evaluations on various flavor and taste indicators of SHLE before and after fermentation, and according to the sensory scoring criteria (Table S1), scores from 0 to 10 were given as quantitative indicators. The grassy, bitter, cassia, sour and fruity flavors in the sensory evaluation indexes were 2% (m/m) lotus leaf solution, 3% (m/m) ginseng solution, 0.3% (m/m) cassia solution, 2 mg/100 mL acetic acid solution and 0.1% (m/m) tangerine peel solution, respectively, used as a 10-point scoring reference, and warm boiled water was used as a 0-point sensory scoring reference. Samples were provided to tasters in single-use plastic cups. The taster only knew the number of the sample. Normal temperature boiled water was used at the beginning of the evaluation and between evaluations of different samples to clean the palate and remove residual flavors.

### 2.14. UPLC-MS-MS Analysis

Each sample (20 mL) was lyophilized and dissolved in 10 mL methanol for 30 min with ultrasonic waves. The samples were mixed completely and subsequently centrifuged at 10,000× *g* for 5 min at 4 °C. Aliquots (1.5 mL) of the supernatants were filtered through a syringe filter with a 0.22 µm nylon membrane and transferred into LC-MS vials. UPLC-MS/MS analysis was carried out using a UPLC IMS Q-TOF (ACQUITY UPLC I-Class Plus/VION IMS Q-Tof, Waters, Massachusetts, USA). The analytical column used was a 2.1 mm ∗ 100 mm, 1.8 μm, ACQUITY UPLC-HSS T3 column (Waters, Massachusetts, MA, USA). Solvent A consisted of water with 0.1% formic acid and solvent B was methanol. The chromatography was carried out at a flow rate of 0.3 mL/min. A linear gradient was programmed for 38 min as follows: 0–3.00 min, 5% B; 3.00–6.25 min, 5% to 20% B; 6.25–27.00 min, 20–50% B; 27.00–30.00 min, 50% to 80% B; 30.00–33.00 min, 80% to 5% B; 33.00–38.00 min, 5% B. The injection volume was 2 µL. The Q-TOF-MS was set for the positive mode and negative mode through a mass range from 50–1000 and a resolution of 5000. The capillary voltage used to record full mass spectra was 0.8 kV. The flow rate of cone gas was 50 L/h, while the flow rate of the desolvation gas (N_2_) was 800 L/h. The desolvation temperature was 550 °C, the ion source temperature was 110 °C, and the collision energies needed to obtain the MS/MS spectra were set from 10 to 30 V.

All data were obtained and processed using Unify 2.0 software. Compound identification was performed by using empirical formula finding, MS/MS library searching, and online database searching. Compound names, peak area, retention time, similarity to metabolites in the database and mass (*m*/*z*) were ultimately imported into Microsoft Excel.

### 2.15. Statistical Analysis

All samples were repeated three times. Statistical analysis was performed using SPSS 22 (Statistical Package for the Social Science, SPSS Ins., Chicago, IL, USA). The results are presented as the means ± SE, and the differences among the different samples were analyzed using one-way analysis of variance (ANOVA, LSD). Correlation and partial correlation were used to analyze the relationship among different factors. Values of *p* < 0.05 or *p* < 0.01 were considered statistically significant.

## 3. Results

### 3.1. LC-MS/MS Analysis of SHLE before and after Fermentation

**Figure 1:**
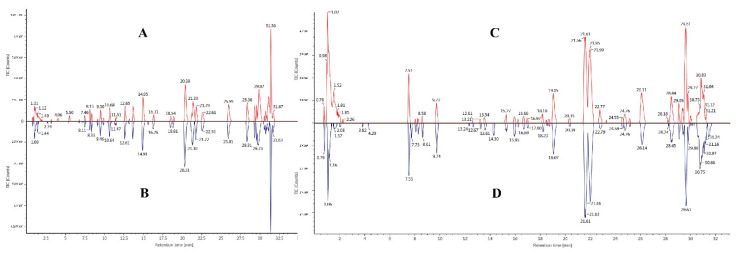
Typical total ion chromatogram (TIC) of SHLE before fermentation ((**A**): positive ion mode; (**C**): negative ion mode) and SHLE after fermentation ((**B**): positive ion mode; (**D**): negative ion mode).

The total ion flow diagram of SHLE samples before and after fermentation in positive and negative ion modes is shown in Figure 1. A total of 50 compounds were identified in positive and negative ion modes (Table S2). After fermentation, 21 kinds of substances were produced, such as dracorhodin, engeletin, 5,7,4′-trihydroxydihydroflavone, 3′,4′,7-trihydroxyflavone, coumestrol and other flavonoids, phenols and other substances. Fermentation makes the original seven substances undetectable, there were 2-ammonio-3-(1H-indol-3-yl) propanoate, poria new acid F, mulberrofuran C, 4,8-Dimethoxyfuro[2,3-b]quinolin-7-ol, phellopterin, epi-cryptoacetalide and 4-*O*-β-d-Glucopyranosyl fagomine.

### 3.2. The Growth of L. fermentum grx08 in SHLE

**Figure 2:**
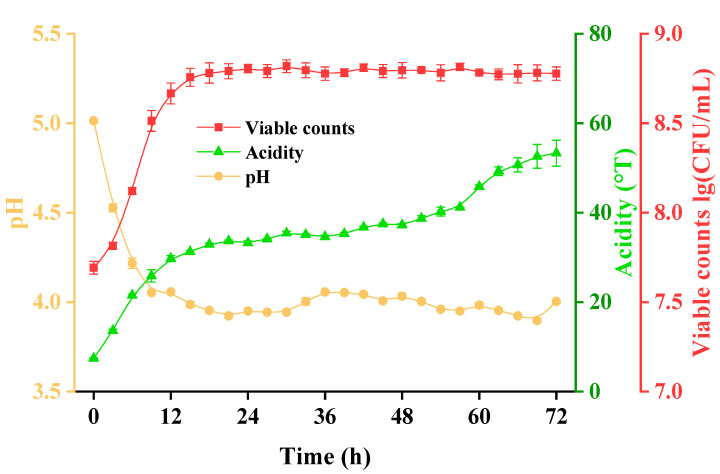
Dynamic changes in pH, acidity and the number of living bacteria during the fermentation of SHLE by *L. fermentum* grx08 for 72 h.

*L. fermentum* grx08 had good growth characteristics in SHLE (Figure 2). After 72 h of fermentation, the lg (CFU/mL) value of living bacteria increased from 7.69 to 8.78, the pH value decreased from 5.01 to 4.00 and the titration acidity increased from 7.42 °T to 53.30 °T. After 18 h of fermentation, the pH value remained basically unchanged but the titration acidity continued to increase and tended to be stable at 72 h.

### 3.3. Effect of L. fermentum grx08 fermentation on Some Active Components of SHLE

**Figure 3:**
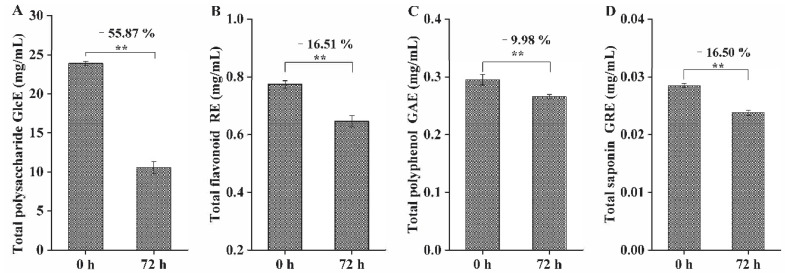
Changes in functional components in SHLE fermented by *L. fermentum* grx08 at 0 h and 72 h. (**A**) Total polysaccharide content (TPSC). Data are expressed as mg of glucose equivalent (GlcE) per mL of SHLE; (**B**) total flavonoid content (TFC). Data are expressed as milligrams of rutin equivalent (RE) per mL of SHLE; (**C**) total polyphenol content (TPC). Data are expressed as mg of gallic acid equivalent (GAE) per mL of SHLE; (**D**) total saponin content (TSC). Data are expressed as mg of ginsenoside Re equivalent (GRE) per mL of SHLE. Data are reported as the mean ± standard error of the mean (SEM). ** indicates that there was a significant difference between 0 h and 72 h (*p* < 0.01).

After fermentation by *L. fermentum* grx08 for 72 h, the TPSC (Figure 3A), TFC (Figure 3B), TPC (Figure 3C) and TSC (Figure 3D) all decreased significantly (*p* < 0.01), of which the TPSC (Figure 3A) decreased the most.

### 3.4. Effect of L. fermentum grx08 fermentation on Volatile Substances in SHLE

**Figure 4:**
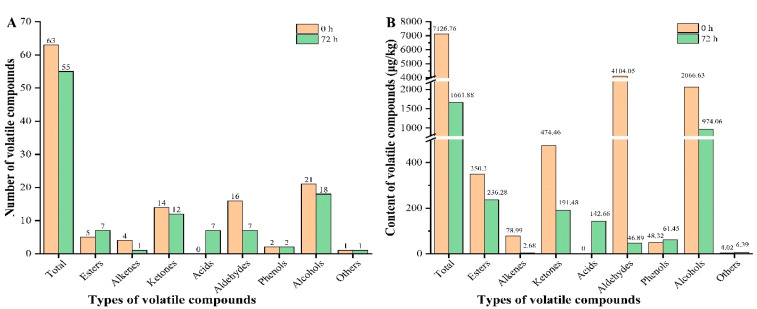
Changes in volatile substances before and after fermentation. (**A**) Number of volatile compounds; (**B**) content of volatile compounds.

The total ion current diagram of SHLE before and after fermentation is shown in Figure S1. The statistical results for volatile compounds (including esters, olefins, ketones, acids, aldehydes, phenols and alcohols, etc.) before and after fermentation were searched by the NIST11 standard library, as shown in Table S3 and Figure 4, and the key flavor substances and odor activity values (OVAs) are shown in Table 2. Both the total number and total content of volatile compounds showed a decreasing trend after fermentation. After fermentation, the number of ester and acid compounds increased.

The contents of acids, phenols and other compounds were higher than those before fermentation; among them, the content of acid substances increased the most (Figure 4B).

After fermentation, the most obvious change was cinnamaldehyde (Figure S1B, RT: 23.3 min), whose content decreased 405.67 times from 3402.57 µg/L to 7.55 µg/L (Table S3 No. 57), and its OAV value decreased from 4.54 to 0.01 (Table 2 No. 6). Therefore, it was no longer the key flavor substance after fermentation. Some aldehydes and alcohols, such as 1-pentanol (Table S3 No. 65), 1-hexanal (Table S3 No. 46) and 1-hexanol (Table S3 No. 67), were undetectable after fermentation. Among all volatile compounds, the OAV value of methyl 2-(methylamino) benzoate (Table 2 No. 1) was the highest before fermentation (1295.36) and after fermentation (884.02). After fermentation, a new alcoholic flavor substance was produced, cinnamyl alcohol (Table 2 No. 17).

Fermentation caused SHLE to also show significant sensory changes (Table 3). Fermentation significantly reduced SHLE’s cinnamon and grassy odors and significantly reduced bitter tastes (*p* < 0.0001). Conversely, fermentation added sour and fruity flavors (*p* < 0.0001).

### 3.5. Effect of L. fermentum grx08 fermentation on the Anti-Obesity Function of SHLE In Vitro

**Figure 5:**
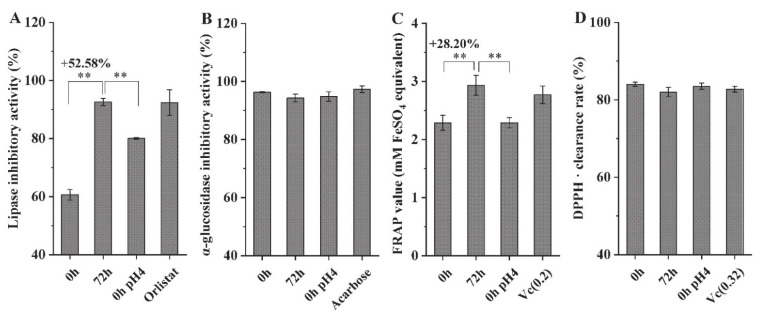
Effects of *L. fermentum* grx08 fermentation on the functional characteristics of SHLE at 0 h and 72 h. (**A**) Inhibition of lipase activity; (**B**) inhibition of α-glucosidase activity; (**C**) total antioxidant capacity (FRAP); (**D**) DPPH· scavenging rate. Data are expressed as the mean ± standard deviation (SEM). ** *p* < 0.01 indicates that there was a significant difference between 0 h and 72 h. FRAP, ferric reducing-antioxidant power. “0 h pH 4” means lactic acid was used to adjust the extraction solution for fermentation for 0 h to the pH value of 4. Positive controls: Orlistat, 0.5 mg/mL. Acarbose, 0.001 mg/mL. Vc(0.2), 0.2 mg/mL vitamin C. Vc(0.32), 0.32 mg/mL vitamin C.

After fermentation for 72 h, the lipase inhibitory activity (Figure 5A) and total antioxidant capacity (Figure 5C) of SHLE were significantly increased. The α-glucosidase inhibitory activity was 94.29% (Figure 5B), the scavenging rate of DPPH· was 81.99% (Figure 5D), and fermentation had no significant effect on either of them (*p* > 0.05).

Pearson correlation analysis (Table 4) showed that the lipase inhibition rate and total antioxidant capacity were significantly positively correlated with acidity and viable bacteria number (*p* < 0.01) and significantly negatively correlated with pH (*p* < 0.01). The inhibition rate of α-glucosidase was significantly correlated with the TPC (*p* < 0.05). The scavenging ability of DPPH· was positively correlated with the TPSC, TFC, TSC and TPC (*p* < 0.01). Partial correlation analysis showed that the lipase inhibition rate was positively correlated with the TFC and TPSC when controlling for the effects of pH and acidity (*p* < 0.05). There was a significant positive correlation between total antioxidant capacity and TPSC when controlling for the influence of acidity (*p* < 0.01).

The pH value of the SHLE was adjusted to 4 (recorded as ‘0 h pH 4’) with lactic acid, which was consistent with the pH value after 72 h of fermentation. Accordingly, after the pH value at 0 h of fermentation of SHLE was adjusted to 4, although the level of lipase activity inhibition and total antioxidant capacity was improved, it was still significantly lower than that at 72 h of fermentation (*p* < 0.01). Adjusting the pH value, similar to fermentation, exhibited no obvious change in α-glucosidase activity inhibition and DPPH· scavenging ability.

**Figure 6:**
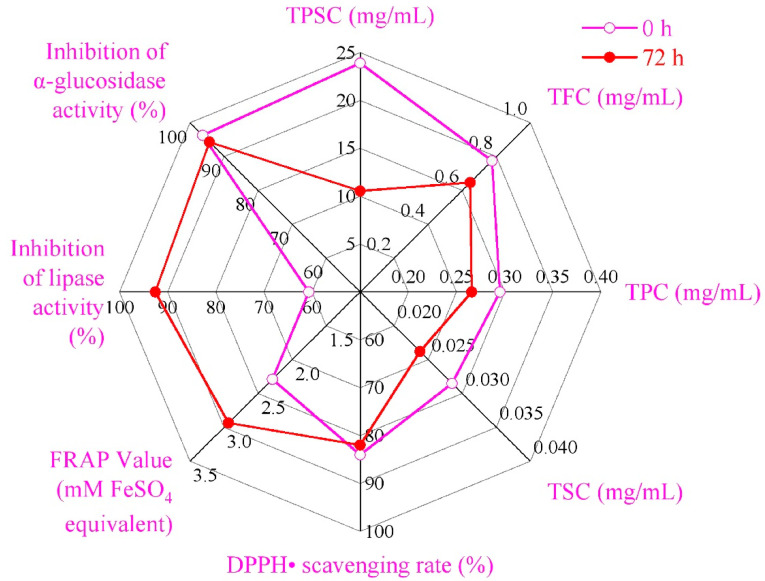
Radar chart of functional components and weight loss efficacy in vitro of SHLE before and after fermentation by *L. fermentum* grx08.

Taken together, as shown in Figure 6, the inhibition of lipase activity and total antioxidant capacity (FRAP value) of SHLE fermented by *L. fermentum* grx08 were significantly enhanced in vitro, although almost all the functional components declined.

## 4. Discussion

In China, “medicine food homology” (MFH) can be used for both food and medicine. In addition to nutritional value, MFH materials also have functions in the prevention and treatment of disease. The use of MFH for dietary regulation will become a new trend. Using renewable resources of MFH to develop and produce new lipid-lowering drugs or health care products that can prevent the occurrence of hyperlipidemia has broad prospects [32]. In recent years, a variety of Chinese herbal medicines have been used to treat obesity and related metabolic disorders [33]. In this study, SHLE was fermented by *L. fermentum* grx08 to explore the effect of fermentation on its flavor and weight loss function. The results indicated that *L. fermentum* grx08 grew well in the SHLE. During the process of fermentation for 72 h, *L. fermentum* grx08 mainly consumed polysaccharides, which may be due to its demand for glycogen. In addition, flavonoids, polyphenols and saponin were utilized to a lesser extent. 

It is encouraging to note that fermentation produced beneficial changes in the flavor of SHLE. First of all, concerning the senses, fermentation increases palatable acidity. The titration acidity increased from 7.42 °T to 53.30 °T after fermentation for 72 h. The results of GC-MS also showed that seven kinds of organic acids were produced after fermentation. The results of these three indicators of sour taste are consistent and can be confirmed by each other. Second, fermentation removes the grassy smell and bitter taste, which may be related to 1-amyl alcohol, 1-hexanal [34] and 1-hexanol [35], which have a bitter taste and grassy smell, which were undetectable after fermentation. In addition, the reduction in bitter taste may also be related to the masking of the sour taste. Third, cinnamaldehyde is the main flavor compound before the fermentation of SHLE, which is also sensory. After fermentation, however, the sensory and OAV values consistently showed the cinnamaldehyde odor as essentially unscented. Finally, the key flavor substances (OAV > 1) after fermentation were only methyl 2-(methylamino)benzoate and linalool. Methyl 2-(methylamino)benzoate is the most important fruit flavor and is widely used in the preparation of orange oil, peach, grape, grapefruit and other flavor substances. Linalool has a bergamot-like aroma. Although the content and OAV value of the above two were reduced after fermentation, the fermented SHLE showed a pleasant fruity aroma without masking the grassy, bitter and cinnamon flavors. In addition, the cinnamyl alcohol produced by fermentation has the aroma of fruit and flowers [36], which also plays an important role in modifying the fruit aroma. Other studies have shown that cinnamyl alcohol can exert anti-obesity effects by inhibiting the increase in PPARγ expression [37].

The main reason for obesity is that energy absorption exceeds consumption, especially related to the excessive absorption of fat and sugar [2]. Therefore, lipase and α-glucosidase inhibitory activities were selected as functional indicators to reduce energy absorption in vitro. The results showed that the lipase inhibition rate of SHLE was significantly increased to 92.58% after 72 h of fermentation with *L. fermentum* grx08, which was similar to the inhibition rate of 95.56% with 0.5 mg/mL orlistat. Correlation analysis showed that the lipase inhibition rate was positively correlated with acidity and the number of living bacteria and negatively correlated with the pH value. This is consistent with the recently reported result that the lipase inhibitory activity of milk fermented by LAB significantly increased [38]. The pH value of SHLE fermented for 0 h was adjusted to 4, which was consistent with the pH value after 72 h of fermentation, and the lipase activity inhibition level was still significantly lower than that of SHLE fermented for 72 h. It is speculated that during the fermentation process, in addition to the organic acid substances that reduce pH value, other substances that can increase the inhibition level of lipase activity are also produced. Partial correlation analysis showed that the lipase inhibition rate was significantly positively correlated with TFC and TPSC by controlling the effects of pH and acidity. Several studies have shown that some flavonoids can inhibit the activity of pancreatic lipase and inhibit the digestion and absorption of dietary fat [39,40]. In addition, flavonoids have different inhibitory effects on pancreatic lipase because they contain different groups. For example, flavonoids containing methoxy groups are well-known as possible natural pancreatic lipase inhibitors [39]. In this study, although fermentation reduced TFC, lipase inhibitory activity increased. This may be due to the production of some new flavonoids with methoxy groups (Table S2), which may have strong inhibitory activity. Studies have shown that natural plant polysaccharides have lipase inhibitory activity [41,42], but they are generally required at higher dosages than flavonoids to have a certain inhibitory effect [42]. α-Glucosidase plays an important role in the digestion and absorption of carbohydrates and is an important target for the control of postprandial blood glucose [43]. Excess sugars can be converted into fat and stored in the body. The inhibition rate of α-glucosidase (94.29%) and the TPC did not change significantly before or after fermentation. Interestingly, correlation analysis showed that there was a significant positive correlation between the α-glucosidase inhibition rate and TPC. A literature review of plant-derived α-glucosidase inhibitors showed that polyphenols exhibited inhibitory activity [43] in vitro.

Obesity and type 2 diabetes are metabolic diseases characterized by inflammation and insulin resistance, which may be partly caused by gram-negative bacterial LPS in the intestine [44]. Systemic inflammation is a key complication of obesity [45], and chronic inflammation and oxidative stress associated with obesity are thought to play a role in promoting obesity-related complications, such as insulin resistance and type 2 diabetes [46]. The total antioxidant capacity of SHLE was improved by fermentation for 72 h. Correlation analysis showed that the total antioxidant capacity was positively correlated with acidity and the number of living bacteria. Fermentation changes the structure of polysaccharides, and the production of new organic acids and other substances may be the reason for the increase in antioxidant capacity [47]. Fermentation slightly reduced the DPPH· scavenging ability of SHLE, which may be related to the decrease in TPSC, TFC, TSC and TPC contents. However, the difference from that before fermentation was not significant. It is possible that the organic acids or other substances produced by fermentation with DPPH· scavenging ability compensated for the reduced DPPH· scavenging ability of the original substances. New substances produced after fermentation, such as dracorhodin [48], engeletin [49], 5,7,4’-trihydroxydihydroflavone [50], 3’,4’,7-trihydroxyflavone [51], and coumestrol [52], have been shown to have antioxidant capacity. In addition, SHLE before and after fermentation had strong DPPH· scavenging ability, which was comparable to 0.32 mg/mL vitamin C (Vc) solution. This may be related to the inclusion of spathulenol (Table S3 No. 88). Studies have shown that spathulenol has antioxidant and anti-inflammatory activities and shows high antioxidant activity in the DPPH system with an IC_50_ of 85.60 µg/mL [53].

## 5. Conclusions

In summary, SHLE was fermented by *L. fermentum* grx08 for 72 h to improve lipase inhibitory activity and total antioxidant capacity, while α-glucosidase inhibitory activity and DPPH· scavenging ability were maintained at a high level, which showed a good overall conditioning effect on many symptoms of obesity in vitro. Although fermentation reduced the content of most functional compounds, at the same time, fermentation also removed the unpleasant flavors of grass, bitterness and cassia and highlighted the fruit flavor so that SHLE fermentation broth not only had a good effect but also a good flavor. Therefore, LAB fermentation of MFH herbal compound extract is a promising strategy for developing antibiotic fat products suitable for long-term use. The daily diet contains many nutritional and nonnutritive ingredients, and their interaction with the SHLE fermentation broth may change the properties determined in vitro. Hence, to develop this fermentation broth into functional foods for weight loss, further studies will be conducted to assess whether these efficacies and activities can be verified in an in vivo model.

## Figures and Tables

**Table 1:** The composition and proportions of SHL.

Latin Scientific Name	Herbal Medicine	Chinese Name	Plant Part	Weight (g)
*Panax ginseng* C. A. Meyer	Ginseng	Renshen	Root	10
*Nelumbo nucifera* Gaertn.	Lotus leaf	Heye	Leaf	6
*Poria cocos* (Schw.) Wolf.	Poria cocos	Fuling	Dry sclerotia	10
*Vigna umbellata* (Thunb.) Ohwi et Ohashi	Rice bean	Chixiaodou	Seed	10
*Citrus sinensis* (Linn.) Osbeck	Tangerine peel	Chenpi	Pericarp	3
*Cinnamomum cassia* Presl	Cassia	Rougui	Cortex	1
Total				40

**Table 2:** Key flavor substances and their OAV values of SHLE before and after fermentation.

No.	Volatile Components	Odor Threshold (μg·L^−^^1^)	OAVs	
			Before Fermentation	After Fermentation
	Ester compounds			
1	Methyl 2-(methylamino)benzoate	0.25	1295.36	884.02
2	Methyl anthranilate	7.00	1.09	0.56
Subtotal	2		2	1
	Ketone compounds			
3	Carvone	27.00	1.42	0.76
Subtotal	1		1	0
	Aldehyde compounds			
4	Butanal, 3-methyl-	0.35	33.81	-
5	Butanal, 2-methyl-	1.00	7.26	-
6	Cinnamaldehyde	750.00	4.54	0.01
7	*n*-Hexanal	73.00	2.36	-
8	Benzene acetaldehyde	6.30	2.10	-
9	Heptanal	4.10	1.85	-
10	Pentanal	12.00	1.49	-
11	Nonanal	8.00	1.27	-
Subtotal	8		8	0
	Alcohol compounds			
12	Linalool	0.22	2181.18	1042.47
13	4-Heptenal, (Z)-	0.03	155.69	-
14	1-Octen-3-ol	1.50	43.37	0.45
15	1-Butanol, 3-methyl-	4.00	3.39	-
16	*n*-Hexanol	5.60	1.93	-
17	Cinnamyl alcohol	77.00	0.00	0.17
Subtotal	6		5	1
Total	17		16	2

OAVs: Odor Activity Values. OAV = Concentration (μg·L^−1^)/Odor threshold(μg·L^−1^). The concentration is shown in Table S3.

**Table 3:** Sensory evaluation.

Sensory Score	Cassia Smell	Grassy Smell	Bitter Taste	Sour Taste	Fruity
Before fermentation	7.40 ± 0.55	8.40 ± 0.55	7.60 ± 1.14	0.40 ± 0.55	0.6 ± 0.55
After fermentation	0.60 ± 0.55 *	0.20 ± 0.45 *	1.00 ± 0.71 *	6.40 ± 1.14 *	5.80 ± 0.84 *

Comparison with the same row before fermentation: * *p* < 0.0001.

**Table 4:** Correlation analysis.

r	Viable Bacteria Number	pH Value	Titrated Acidity	TPSC	TFC	TPC	TSC
lipase inhibition rate	0.881 **	−0.764 **	0.928 **	−0.757 **	−0.757 **	−0.602 **	−0.811 **
α-glucosidase inhibition rate	−0.642 **	0.674 **	−0.343	0.140	0.179	0.477 *	−0.023
total antioxidant capacity	0.904 **	−0.917 **	0.842 **	−0.627 **	−0.684 **	−0.676 **	−0.713 **
DPPH· scavenging ability	−0.810 **	0.700 **	−0.725 **	0.539 **	0.646 **	0.460 **	0.625 **
lipase inhibition rate ^#^	0.722 **	-	-	0.804 **	0.449 *	−0.442 *	−0.202
total antioxidant capacity ^#^	0.683 **	−0.754 **	-	0.665 **	0.286	−0.535 **	−0.013

r: correlation coefficient; * *p* < 0.05, ** *p* < 0.01. ^#^: Partial correlation analysis. ‘-’ control variable. TPSC: total polysaccharides content. TFC: total flavonoids content. TPC: total polyphenols content. TSC: total saponin content.

## Data Availability

Not applicable.

## Supplementary Materials

The following are available online at www.mdpi.com/article/10.3390/foods11091221/s1. Figure S1: GC-MS total ion chromatogram before and after fermentation (A) after fermentation (B) before fermentation. Table S1: Sensory Scoring Criteria. Table S2: Compounds were identified in SHLE before and after fermentation by UPLC-MS-MS. Table S3: Changes of relative mass concentration of volatile flavor compounds before and after fermentation.
