# Supplementary material for: In Vitro Anti-Obesity Effect of Shenheling Extract (SHLE) Fermented with Lactobacillus fermentum grx08
Source: Foods. 2022 Apr 23;11(9):1221. doi: 10.3390/foods11091221 (PMC9104015; doi:10.3390/foods11091221)
Supplement: Supplementary file 1 [file foods-11-01221-s001.zip › foods-1621114-supplementary.pdf]

Supplementary data

Table S1 Sensory Scoring Criteria

Evaluation indicators	Scoring reference standard			Score
Grassy	0 points: plain water	5 points: 1% (m/m) lotus leaf solution	10 points: 2% (m/m) lotus leaf solution	
Bitterness	0 points: plain water	5 points: 1.5% (m/m) ginseng solution	10 points: 3% (m/m) ginseng solution	
Cassia	0 points: plain water	5 points: 0.15% (m/m) cassia solution	10 points: 0.3% (m/m) cassia solution	
Sour taste	0 points: plain water	5 points: 1 mg/100mL acetic acid solution	10 points: 2 mg/100mL acetic acid solution	
Fruit flavor	0 points: plain water	5 points: 0.05% (m/m) tangerine peel solution	10 points: 0.1% (m/m) tangerine peel solution	

Table S2. Compounds were identified in SHLE before and after fermentation

Neg.ion									
**No.**	**Component name**	**Observed RT (min)**	**Neutral mass (Da)**	**Observed neutral mass (Da)**	**Observed m/z**	**Mass error (mDa)**	**Molecular ions/ adduct ions**	**Before fermentation**	**After fermentation**
1	5-methoxy-6-methyl-2-phenylchromen-7-one	4.29	266.09429	266.0976	265.0903	3.3	-H		+
2	(-) - epicatechin-5-o- β- D-glucopyranoside	7.53	452.13186	452.1272	451.1199	-4.7	-H		+

3	2-Ammonio-3-(1H-indol-3-yl) propanoate (Tryptophan)	8.22	204.08988	204.0865	203.0792	-3.4	-H	+	
4	3,4-dimethoxybenzoic acid	8.59	182.05791	182.055	181.0477	-2.9	-H	+	+
5	(2R, 3R) - 3,5,7,3', 5' - pentahydroxyflavane	9.74	290.07904	290.0764	289.0691	-2.7	-H		+
6	8-(Δ 2-isopentenyl) - 5,7,3', 4' - tetrahydroxyflavone	14.29	354.11034	354.1055	353.0982	-4.8	-H		+
7	1 - (2-hydroxy-5-anisole) - ethanone	14.3	166.06299	166.0621	165.0548	-0.9	-H		+
8	1-(4-hydroxy-3-methoxyphenyl)-Ethanone	14.3	166.06299	166.0621	165.0548	-0.9	-H		+
9	7,4′,7′′,4′′′-Tetra-O-methylamantoflavin	15.28	594.1526	594.1526	593.1453	0	-H		+
10	5-ethenyl-4-(hydroxymethyl)-3,4-dihydropyrano[3,4-c]pyridin-1-one	15.94	205.07389	205.0727	204.0655	-1.2	-H		+
11	(2R,3R)-5,7-dihydroxy-2-(4-hydroxyphenyl)-3-[(2S,3R,4R,5S,6S)-3,4,5-trihydroxy-6-methyl-oxan-2-yl]oxy-chroman-4-one	16.7	434.1213	434.1182	433.1109	-3.1	-H		+
12	Atractylodes aglycone 2-O-β-D-glucoside	18.24	482.25158	482.2483	481.241	-3.3	-H		+
13	(2R)-2-[(2R,3R,3aR,6S,7S,9bR)-6-(2-Carboxyethyl)-2-hydroxy-7-isopropenyl-3a,6,9b-trimethyl-2,3,3a,4,6,7,8,9b-octahydro-1H-cyclopenta[a]naphthalen-3-yl]-6-methyl-5-methyleneheptanoic acid (Poria New Acid F)	18.7	498.33452	498.3327	497.3254	-1.8	-H	+	
14	Mulberrofuran C	20.32	580.17333	580.1716	579.1643	-1.7	-H	+	
15	nobiletin- 3 - O -β-D-glucoside	20.36	580.17921	580.1774	579.1701	-1.9	-H		+
16	Quercetin-3-o- α- D-glucuronide	21.58	478.07474	478.0712	477.0639	-3.5	-H		+
17	Myricetin-3-o- α- L-rhamnopyranoside	22.02	464.09548	464.0927	463.0854	-2.8	-H		+
18	Kaempferol-3,7-di-o- β- D-glucoside	22.18	610.15338	610.1527	609.1454	-0.7	-H		+
19	Kaempferol-3-o- β- D-glucuronic acid	24.59	462.07983	462.0773	461.0701	-2.5	-H		+

20	Luteolin-7-o- β- D-glucopyranoside	24.77	448.10056	448.0981	447.0908	-2.5	-H		+
21	6-Methoxykaempferol-3-O-β-D-galactopyranoside	25.16	478.11113	478.1086	477.1013	-2.6	-H		+
22	2',3,4',5,7-pentahydroxyflavone (Quercetin)	28.34	302.04265	302.0383	301.031	-4.4	-H	+	+
23	Deacetylnomilinic acid	30.12	490.22028	490.2186	489.2114	-1.6	-H		+
24	Sanleng acid	31.04	330.24062	330.2356	329.2284	-5	-H	+	+
**Pos.ion**									
**No.**	**Component name**	**Observed RT (min)**	**Neutral mass (Da)**	**Observed neutral mass (Da)**	**Observed m/z**	**Mass error (mDa)**	**Molecular ions/ adduct ions**	**Before fermentatio**	**After fermentation**
1	4,8-Dimethoxyfuro[2,3-b]quinolin-7-ol	4.05	245.06881	245.0647	268.054	-4.1	+Na	+	
2	Crotepoxide	6.76	362.10017	362.1025	363.1097	2.3	+H	+	+
3	Coixtrin	8.15	165.04259	165.0465	188.0357	3.9	+Na	+	+
4	5,7,4 '- trihydroxydihydroflavone	8.34	272.06847	272.072	273.0793	3.5	+H		+
5	3 ', 4', 7-trihydroxyflavone	9.14	270.05282	270.0566	271.0638	3.7	+H		+
6	2 ', 7-dihydroxy-4', 5 '- dimethoxyisoflavone	9.5	314.07904	314.0757	315.083	-3.3	+H	+	+
7	1,2-dimethoxy-dibenzo[de,g]quinolin-7-one	9.51	291.08954	291.0908	314.08	1.2	+Na	+	+
8	3,9-Dihydroxycoumestan	9.6	268.03717	268.0417	291.0309	4.5	+Na		+
9	8-(2,3-Dihydroxy-3-methylbutyl)-7-methoxy-2H-chromen-2-one	10.65	278.11542	278.116	301.1052	0.6	+Na		+
10	hesperetin 7-O-neohesperoside	11.41	610.18977	610.1901	611.1974	0.4	+H	+	+
11	3'-Deoxysappanone B	12.66	314.11542	314.1121	315.1194	-3.3	+H	+	+
12	4-methoxy-9-(3-methylbut-2-enoxy)furo[3,2-g]chromen-7-one (Phellopterin)	13.69	300.09977	300.0991	301.1063	-0.7	+H	+	

13	7-methyl-6,7-dihydro-5H-benzo<g>-1,3-benzodioxolo<6,5,4-de>quinoline	13.7	277.11028	277.1143	300.1036	4.1	+Na	+	+
14	6-hydroxy-2 - [2 - (4-hydroxyphenyl) ethyl] chromone	14.97	282.08921	282.0921	283.0994	2.9	+H	+	+
15	7β-(3-Ethyl-cis-crotonoyloxy)-1α-(2-methyl-butyryloxy)-3,14-dehydro-Z-notonipetranone	18.53	430.27192	430.2695	453.2588	-2.4	+Na	+	+
16	6-hydroxy-2 - [2 - (4 '- methoxyphenyl) ethyl] chromone	20.39	296.10486	296.1054	297.1127	0.5	+H	+	+
17	Nigakilactone G	29.3	474.22537	474.2291	475.2364	3.7	+H	+	+
18	2'-Methoxykurarinone	29.3	452.21989	452.2216	453.2289	1.7	+H		+
19	Salviamiltamide	20.39	295.10559	295.1025	296.1098	-3.1	+H	+	+
20	lamiophomiol B	20.39	242.07904	242.084	265.0732	5	+Na	+	+
21	laminitol	29.86	372.04813	372.0522	373.0594	4	+H, +Na	+	+
22	(2R,4R)-4,6',6'-Trimethyl-4,5,6',7',8',9'-hexahydro-1'H,3H-spiro[furan-2,3'-naphtho[1,2-c]furan]-1'-one (Epi-Cryptoacetalide)	31.07	286.15689	286.1573	309.1465	0.4	+Na	+	
23	4-O-β-D-Glucopyranosyl fagomine	31.08	309.14237	309.1423	310.1496	-0.1	+H	+	
24	(1R)-1,6-dimethyl-1,2-dihydronaphtho[1,2-g][1]benzofuran-10,11-dione	31.1	278.09429	278.096	301.0852	1.7	+Na	+	+
25	Neociwujiaphenol	31.37	402.16785	402.1716	403.1789	3.8	+H	+	+
26	1H-2,6-Dioxacyclopent[cd]inden-1-one, 4-[(acetyloxy)methyl]-5-(hexopyranosyloxy)-2a,4a,5,7b-tetrahydro-	31.78	414.11621	414.1211	437.1103	4.9	+Na		+

The '+' in the columns before fermentation and after fermentation indicates that the compound had been detected.

Table S3 Changes of relative mass concentration of volatile flavor compounds before and after fermentation

No.	RT (min)	CAS #	Volatile Components	Relative mass concentration （μg·L^{-1}） Before fermentation	After fermentation
			Ester compounds		
1	2.38	141-78-6	Ethyl Acetate	3.66	-
2	25.1	134-20-3	Methyl anthranilate	7.65	3.89
3	26.89	85-91-6	Methyl 2-(methylamino)benzoate	323.84	221.00
4	28.14	103-54-8	2-Propen-1-ol,3-phenyl-, 1-acetate	9.01	4.68
5	30.32	17092-92-1	(2,6,6-Trimethyl-2-hydroxycyclohexylidene)acetic acid lactone	-	1.34
6	30.41	109-20-6	Geranyl isovalerate	-	0.56
7	30.71	57156-91-9	2,5-Octadecadiynoic acid, methyl ester	-	0.48
8	34.4	120-51-4	Benzyl Benzoate	6.14	4.32
		Subtotal	8	5	7
			Alkene compounds		
9	15.08	586-62-9	Cyclohexene,1-methyl-4-(1-methylethylidene)-	2.53	-
10	15.34	460-01-5	(3E,5E)-2,6-Dimethyl-1,3,5,7-octatetrene	11.13	-
11	18.91	6090-09-1	4-acetyl-1-methyl-1-cyclohexene	12.18	2.68
12	19.11	1195-92-2	limonene 1,2-epoxide	53.14	-
		Subtotal	4	4	1
			Ketone compounds		

13	3.38	6137-11-7	4-methylheptan-3-one	-	1.97
14	7.54	644-78-0	2-Hydroxychalcone	-	0.66
15	10.46	110-43-0	2-Heptanone	7.11	5.09
16	14.03	110-93-0	5-Hepten-2-one, 6-methyl-	45.51	-
17	16.77	98-86-2	Acetophenone	4.84	-
18	16.95	38284-27-4	3,5-Octadien-2-one	18.68	-
19	17.53	15932-80-6	Cyclohexanone, 5-methyl-2-(1-methylethylidene)-	2.54	-
20	17.74	30086-02-3	3,5-Octadien-2-one, (E,E)-	6.34	-
21	19.42	464-49-3	(R)-camphor	246.68	109.10
22	19.89	30460-92-5	Pinocarvone	-	1.75
23	20.05	528-21-2	Gallacetophenone	6.31	-
24	21.19	5948-04-9	Cyclohexanone,2-methyl-5-(1-methyl ethenyl)-, (2R,5R)-rel-	79.31	29.30
25	22.36	99-49-0	Carvone	38.25	20.53
26	22.65	89-81-6	2-Cyclohexen-1-one,3-methyl-6-(1-m ethylethyl)-	9.39	12.27
27	23.08	16750-82-6	(S)-3-Methyl-6β-isopropenyl-2-cyclo hexene-1-one	-	2.61
28	25.05	491-09-8	2-Cyclohexen-1-one, 3-methyl-6-(1-methylethylidene)-	5.07	-
29	26.1	23696-85-7	2-Buten-1-one, 1-(2,6,6-trimethyl-1,3-cyclohexadien-1-yl)-	-	4.10
30	29.09	14901-07-6	3-Buten-2-one, 4-(2,6,6-trimethyl-1-cyclohexen-1-yl)	-	3.08

			-		
31	29.11	79-77-6	β-Ionone	1.82	-
32	29.18	23267-57-4	3-Buten-2-one, 4-(2,2,6-trimethyl-7-oxabicyclo[4.1.0]hept-1-yl)-	2.62	1.01
		Subtotal	20	14	12
			Acid compounds		
33	2.44	64-19-7	Acetic acid	-	123.78
34	14.45	142-62-1	Hexanoic acid	-	14.36
35	17.44	111-14-8	Heptanoic acid	-	1.71
36	18.49	1783-84-2	8,11,14-Eicosatrienoic acid, (Z,Z,Z)-	-	0.60
37	18.6	1883-13-2	Dodecanoic acid, 3-hydroxy-	-	0.55
38	19.97	7333-25-7	10,12-Octadecadiynoic acid	-	1.05
39	29.56	2507-55-3	Tetradecanoic acid, 2-hydroxy-	-	0.61
		Subtotal	7	0	7
			Aldehyde compounds		
40	2.89	590-86-3	Butanal, 3-methyl-	11.83	-
41	3.02	96-17-3	Butanal, 2-methyl-	7.26	-
42	3.61	110-62-3	Pentanal	17.88	-
43	5.28	1576-87-0	2-Pentenal, (E)-	2.73	-
44	6.35	107-86-8	2-Butenal, 3-methyl-	-	1.43
45	6.48	5204-80-8	4-Pentenal, 2-ethyl-	-	0.28
46	6.94	66-25-1	1-Hexanal	172.42	-
47	8.19	98-01-1	Furfural	4.05	-
48	9.04	6728-26-3	2-Hexenal, (E)-	9.83	-
49	10.94	111-71-7	Heptanal	7.58	-

50	13.12	100-52-7	Benzaldehyde	266.16	13.03
51	14.99	4313-03-5	2,4-Heptadienal, (E,E)-	5.37	-
52	16.05	122-78-1	Benzeneacetaldehyde	13.26	-
53	18.1	124-19-6	Nonanal	10.16	-
54	19.9	104-53-0	Benzenepropanal	49.55	-
55	21.43	99172-18-6	3,5-Heptadienal, 2-ethylidene-6-methyl-	-	0.36
56	21.51	15764-16-6	Benzaldehyde, 2,4-dimethyl-	113.29	23.27
57	23.25	104-55-2	Cinnamaldehyde	3402.57	7.55
58	30.43	1504-74-1	2-Propenal, 3-(2-methoxyphenyl)-	10.11	-
59	34.19	1620-98-0	3,5-di-tert-Butyl-4-hydroxybenzaldehyde	-	0.98
		Subtotal	20	16	7
			Phenolic compounds		
60	23.83	89-83-8	Thymol	26.29	37.05
61	29.71	128-37-0	Butylated Hydroxytoluene	22.02	24.40
		Subtotal	2	2	2
			Alcohol compounds		
62	1.48	64-17-5	Ethanol	16.73	71.57
63	3.35	616-25-1	1-Penten-3-ol	16.46	-
64	4.69	123-51-3	1-Butanol, 3-methyl-	13.57	-
65	5.82	71-41-0	1-Pentanol	8.43	-
66	5.9	1576-95-0	2-Penten-1-ol, (Z)-	7.03	-
67	9.79	111-27-3	1-Hexanol	10.78	-
68	10.01	111-70-6	1-Heptanol	0.50	-
69	10.1	928-96-1	3-Hexen-1-ol, (Z)-	3.89	2.22

70	10.75	928-97-2	3-Hexen-1-ol, (E)-	-	0.57
71	10.84	6728-31-0	4-Heptenal, (Z)-	3.89	-
72	10.94	51174-44-8	4-Penten-1-ol, 3-methyl-	-	0.70
73	11.4	626-89-1	1-Pentanol, 4-methyl-	-	0.48
74	14.56	3391-86-4	1-Octen-3-ol	65.05	0.67
75	14.68	99-48-9	Carveol	20.75	-
76	17.51	5989-33-3	2-Furanmethanol,5-ethenyltetrahydro -a,a,5-trimethyl-, (2R,5S)-rel-	-	10.85
77	17.98	78-70-6	Linalool	479.86	229.34
78	18.69	7212-40-0	2-Cyclohexen-1-ol, 1-methyl-4-(1-methylethenyl)-, trans-	25.34	6.59
79	18.73	29803-81-4	2-Cyclohexen-1-ol, 1-methyl-4-(1-methylethyl)-, trans-	9.13	-
80	19.17	3886-78-0	cis-p-Mentha-2,8-dien-1-ol	1.58	6.17
81	19.59	6627-74-3	2-Cyclohexene-1-methanol, 2,6, 6-trimethyl-	-	2.51
82	20.25	507-70-0	endo-Borneol	25.59	-
83	20.49	562-74-3	Terpinen-4-ol	574.29	279.83
84	20.74	1197-01-9	Benzenemethanol,α,α,4-trimethyl-	31.39	10.50
85	20.98	98-55-5	α-Terpineol	607.59	310.14
86	22.52	1197-06-4	2-Cyclohexen-1-ol, 2-methyl-5-(1-methylethenyl)-, (1R,5R)-rel-	119.21	16.58
87	25.59	104-54-1	Cinnamyl alcohol	-	12.87
88	31.42	6750-60-3	Spathulenol	25.58	11.59
89	32.56	117591-80-7	Ginsenol	-	0.86

		Subtotal	28	21	18
			Other compounds		
90	17.59	1124-20-5	1-methyl-3-prop-1-en-2-ylbenzene	-	6.39
91	28.6	719-22-2	2,5-Cyclohexadiene-1,4-dione, 2,6-bis(1,1-dimethylethyl)-	4.02	-
		Subtotal	2	1	1
		Total	91	63	55

“-”means not detected.
